# Bibliometric review of 1992–2022 publications on acupuncture for cognitive impairment

**DOI:** 10.3389/fneur.2022.1006830

**Published:** 2022-09-26

**Authors:** Runjin Zhou, Lu Xiao, Wei Xiao, Yanfei Yi, Huanhuan Wen, Hongda Wang

**Affiliations:** ^1^Medical College of Acupuncture-Moxibustion and Rehabilitation, Guangzhou University of Chinese Medicine, Guangzhou, China; ^2^Ganzhou Cancer Hospital, Ganzhou, China

**Keywords:** acupuncture therapy, cognitive impairment, VOSviewer, CiteSpace, scientometric analysis

## Abstract

**Objective:**

To explore the development context, research hotspots, and frontiers of acupuncture therapy for cognitive impairment (CI) from 1992 to 2022 by visualization analysis.

**Methods:**

Articles about acupuncture therapy for cognitive impairment were retrieved from the Web of Science Core Collection (WoSCC) until 1 March 2022. Basic information was collected by Excel 2007, and VOSviewer 1.6.17 was used to analyze the co-occurrence of countries, institutes, and authors. Co-citation maps of authors and references were analyzed by CiteSpace V.5.8.R3. In addition, CiteSpace was used to analyze keyword clusters and forecast research frontiers.

**Results:**

A total of 279 articles were retrieved, including articles from 19 countries, 334 research institutes, and 101 academic journals. The most published country and institutes were the People's Republic of China (217) and the Fujian University of Traditional Chinese Medicine (40). Ronald C Petersen owned the highest co-citations (56). Keywords and co-cited references cluster showed the main research directions in this area, including “ischemic stroke,” “cerebral ischemia/reperfusion,” “mild cognitive impairment,” “Alzheimer's disease,” “vascular dementia,” “vascular cognitive impairment with no dementia,” “multi-infarct dementia,” “synaptic injury,” “functional MRI,” “glucose metabolism,” “NMDA,” “nuclear factor-kappa b pathway,” “neurotrophic factor,” “matrix metalloproteinase-2 (MMP-2),” “tumor necrosis factor-alpha,” “Bax,” “Caspase-3,” and “Noxa”. Trending keywords may indicate frontier topics, such as “randomized controlled trial,” “rat model,” and “meta-analysis.”

**Conclusion:**

This research provides valuable information for the study of acupuncture. Diseases focus on mild cognitive impairment (MCI), Alzheimer's disease (AD), and vascular dementia (VaD). Tauopathies with hyperphosphorylation of Tau protein as the main lesions also need to be paid attention to. The development of functional magnetic resonance imaging (fMRI) will better explain the therapeutic effect of acupuncture treatment. The effect of acupuncture on a single point is more convincing, and acupuncture on Baihui (GV20) may be needed in the future. Finally, the implementation of high-quality multicenter randomized controlled trials (RCTs) requires increased collaboration among experts from multiple fields and countries.

## Introduction

Cognitive function refers to all kinds of conscious mental activities that human beings always have in the state of awakening, such as simple determination, perception, understanding, and judgment of themselves and the environment to complete complex mathematical calculations (1, 2). Cognitive impairment (CI) is a pathological process in which learning, memory, and thinking judgment related to the brain's advanced intelligent processing are abnormal, resulting in learning and memory disorders, accompanied by aphasia, apraxia, agnosia, and other changes, from mild cognitive impairment (MCI) to dementia (1, 2). MCI is a symptomatic diagnosis in which a patient has a memory or cognitive dysfunction that does not significantly affect daily functioning and does not reach the level of dementia (3). It is an intermediate state between normal aging and dementia, and the prevalence of MCI in adults 65 years and older ranges from 3 to 20%, with more than half progressing to dementia within 5 years (4, 5). Dementia is an acquired intelligence impairment syndrome with cognitive impairment as its core symptom. Cognitive impairment involves memory, learning, orientation, understanding, judgment, computation, language, visual space, and other functions (6). At present, the international diagnosis of dementia includes the American Diagnostic and Statistical Manual of Mental Disorders (DSM-V) (1) and the International Classification of Diseases, 11th Revision (ICD-11) (2). According to the lesion location, it can be divided into cortical dementia (Alzheimer's disease and frontotemporal degeneration), subcortical dementia (vascular dementia [VaD]), mixed cortical and subcortical dementia (multiple infarct dementia, infectious dementia, poisoning, and metabolic encephalopathy), and other dementias (post-traumatic brain injury dementia, etc.).

Of these, Alzheimer's disease (AD) has the highest incidence, and the number of patients in the world has reached 50 million, which is expected to increase to 152 million in 2050, according to the World Alzheimer Report 2019. Patients with AD have decreased the ability of daily living and abnormal mental behavior, posing a significant burden on family caregivers and society (7). It is necessary to find an effective, safe, and inexpensive treatment for older patients. In 2019, the 72nd World Health Assembly passed the ICD-11, which includes traditional Chinese medicine for the first time. Acupuncture is widely used in Asia as one of the non-pharmacological interventions of traditional Chinese medicine. The results of case-control trials based on functional magnetic resonance imaging (fMRI) showed that acupuncture at the Taixi (KI3) acupoint could activate neurons in the cerebral cortex related to cognition, providing imaging evidence support for clinical treatment of CI (8, 9). Randomized controlled trials (RCTs) suggested that acupuncture could improve cognitive function in patients with mild to moderate cognitive impairment (10, 11). In addition, animal experiments are trying to clarify the mechanism of acupuncture (12, 13).

Acupuncture is a technique that has been practiced for thousands of years, and its description can be traced back to a book called *The Huangdi's Internal Classic*, dating from the Han Dynasty (14). During the operation, Deqi is generated by needles into specific acupoints of the human body, so as to achieve the effect of treating diseases (15).

In recent years, more and more research articles on acupuncture have been published. Currently, the reporting quality of RCTs of acupuncture for MCI is moderate to low (16, 17). Most clinical trials did not mention allocation concealment and blinding, nor did they strictly followed the Consolidated Standards for Reporting of Trials (CONSORT) statement and Standards for Reporting Interventions in Controlled Trials of Acupuncture (STRICTA). There is currently a lack of systematic review of trends in this field. Bibliometric visualization analysis is a quantitative analysis method that combines mathematics and statistical methods. It can intuitively highlight the quantitative characteristics of research articles in a certain field and help researchers grasp the development characteristics of the field over time (18). Therefore, this study adopts bibliometric analysis to conduct a systematic review of the application of acupuncture therapy to CI research, aiming to understand the cooperative network, and evaluate the research trends and frontiers.

## Materials and methods

### Data source and search strategy

Article retrieval was conducted on the Science Citation Index-Expanded (SCI-E) of the Web of Science Core Collection (WoSCC) on 20 March 2022. WoSCC is a relatively comprehensive citation database, and considering that it has the highest applicability with CiteSpace software, so WoSCC was chosen as the preferred retrieval database in this study. The terms “Acupuncture” and “Cognitive Impairment” were used in the MeSH (https://www.ncbi.nlm.nih.gov/mesh) search. The data retrieval strategy is as follows: TS = (Acupuncture OR Pharmacopuncture OR Acupressure OR Acupuncture Therapy OR Acupuncture Point^*^ OR acupunct^*^ OR needl^*^ OR Electroacupuncture OR Ear Acupuncture OR Auricular^*^ OR meridian^*^ OR acupoint^*^) AND TS= (Cognitive Dysfunction OR Cognitive Impairment OR Neurocognitive Disorder OR Cognitive Decline OR Mild cognitive impairment OR Alzheimer's disease OR dementia OR Vascular dementia). The time span: 01-01-1992 to 01-03-2022.

### Inclusion/exclusion criteria

#### Inclusion criteria

The article type is mainly articles in English, and the research type includes randomized controlled trials (RCTs), retrospective studies, case reports, and animal experiments. The main study population is patients with CI, and the intervention is acupuncture therapy.

#### Exclusion criteria

Secondary article, such as reviews and meta-analyses, were excluded. In addition, book chapters, letters, editorial material, and meeting abstracts were excluded. At the same time, by reading the abstracts and full texts, researchers should also exclude articles that only briefly mention acupuncture therapy without involving specific therapeutic effects and mechanisms.

### Data collection

**Figure 1:**
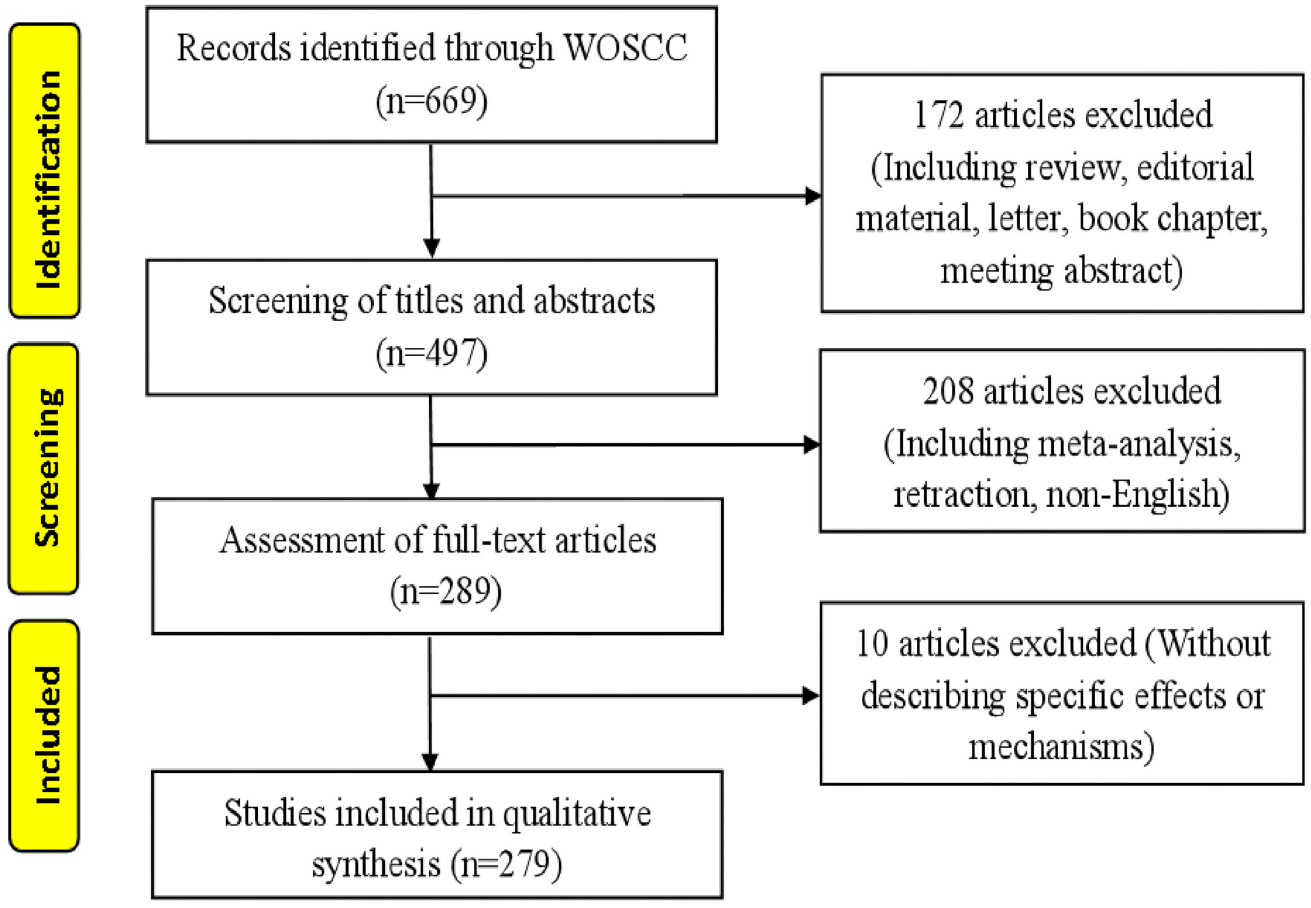
Articles screening flow chart. Two researchers independently evaluated 669 articles according to the inclusion and exclusion criteria and finally obtained 279 qualified articles.

Raw data from WoSCC were downloaded and verified by two members (LX and RJZ), respectively. The flow chart of research inclusion is shown in Figure 1. The data were then imported into CiteSpace V.5.8.R3 (Drexel University, Philadelphia, PA, USA) and VOSviewer 1.6.17 (Leiden University, Van Eck NJ). The information generated by the software is imported into Excel 2007 (Redmond, WA, USA).

### Statistical methods

The WoSCC database was used to analyze the characteristics of the article, such as the number of annual publications and citations, journal sources, research areas, H-index, and impact factor. VOSviewer is a bibliometric analysis software jointly developed by Nees Jan van Eck and Ludo Waltman for mapping scientific knowledge (19). It was used to analyze the co-occurrence of countries/regions, institutes, and authors (which were based on the first author). There were three types of mapping generated: Network Visualization, Overlay Visualization, and Density Visualization. For the Density Visualization, darker yellow indicated more important research. CiteSpace is a tool for visualizing and analyzing trends and patterns in scientific articles (20, 21). It was used for reference/author co-citation analysis, we define that the nodes in the co-citation knowledge graph represent different documents, and the size of the node is proportional to the number of references cited in a specific period. Similarly, a connection between nodes indicates the degree of relationship, the thicker the line, the stronger the connection. Co-occurrence of keywords was analyzed by CiteSpace. We analyzed the characteristics related to keyword clusters, in which the purple reference ring represented the research's high mediating centrality, which played a role in connecting various documents, and the orange represented the newly emerging research (22). This will make it more intuitive to observe the trend of various research hotspots over time.

## Results

### Annual publications and citations

**Figure 2:**
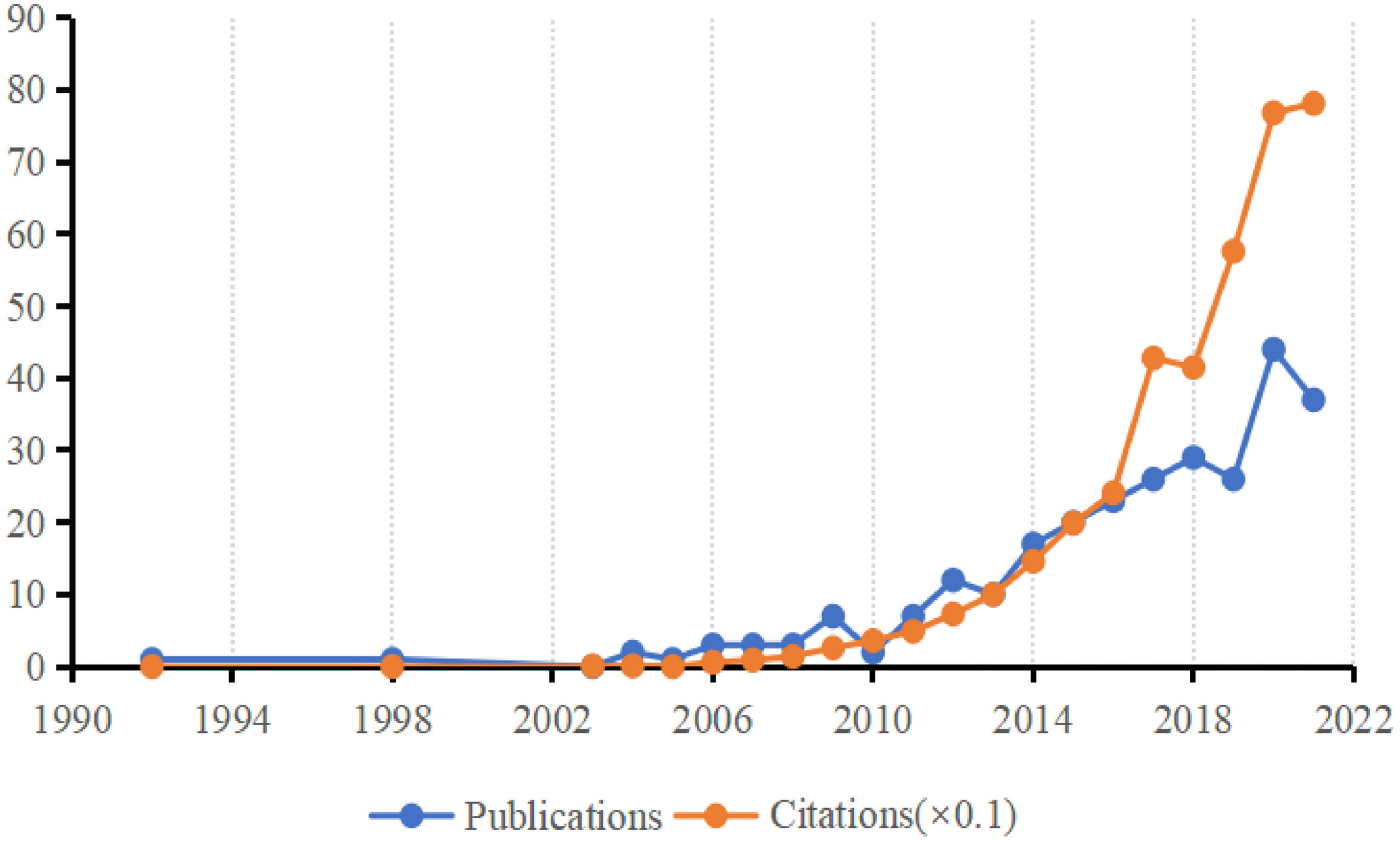
Sum of publications and citations. The number of publications and citations from 1992 to 2021 is described by a line chart, where blue represent the number of publications and yellow represent the number of citations (× 0.1).

In total, 279 articles were included from 1992 (*n* = 1) to 2021 (*n* = 37), the citations of these articles also increased rapidly from 2003 (*n* = 1) to 2021 (*n* =781), with a total of 3,869 citations (Figure 2).

### Research areas analysis

**Figure 3:**
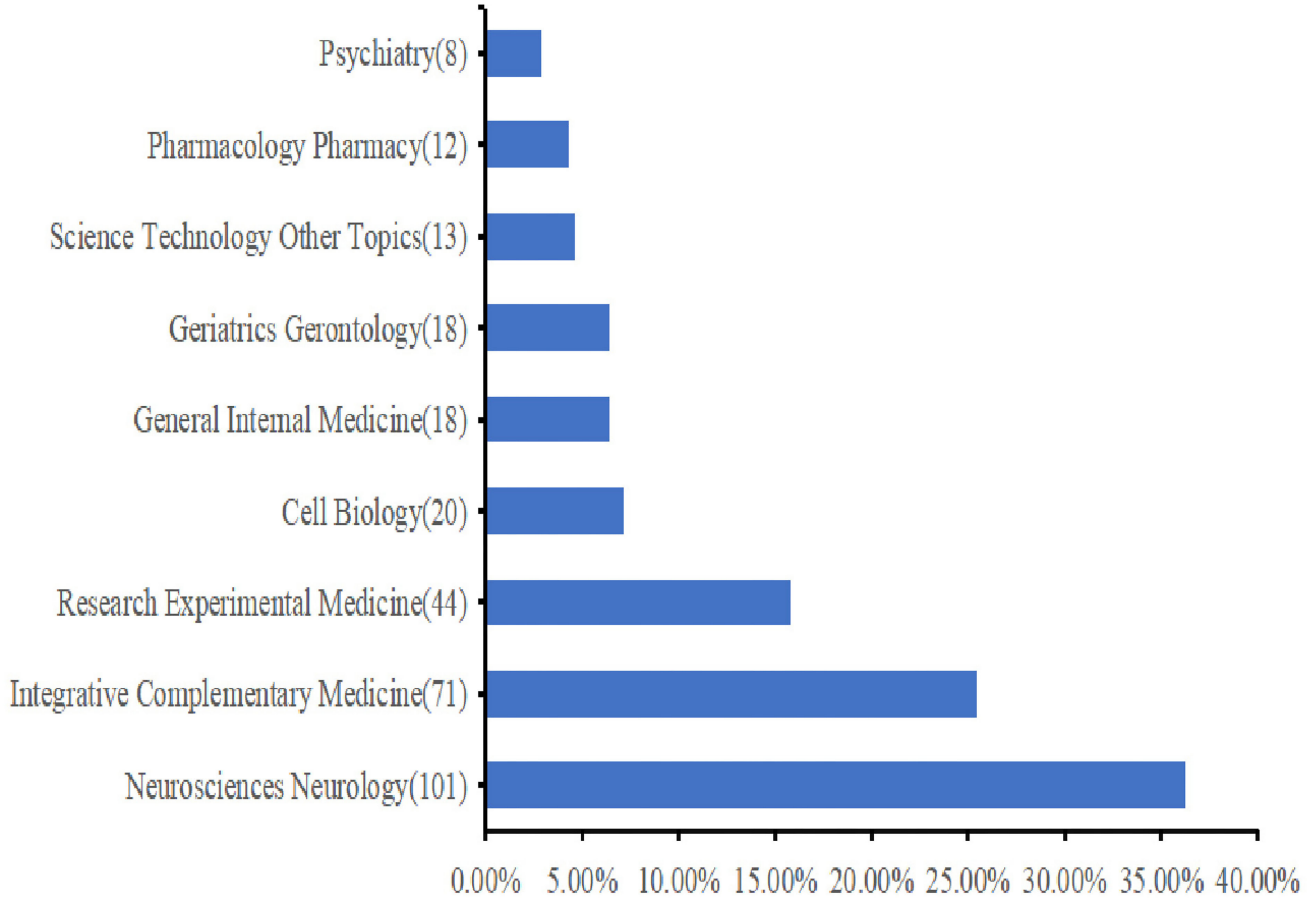
The top 10 research areas about acupuncture therapy for cognitive impairment (CI). The bar chart is used to describe the top 10 research areas. Each specific research area is listed on the *Y*-axis, and the corresponding percentage on the *X*-axis has its proportion.

A total of 33 research areas were represented. Neurosciences and Neurology (*n* = 101), Integrative Complementary Medicine (*n* = 71), and Research Experimental Medicine (*n* = 44) occupied the main position. Figure 3 shows the top 10 research areas in acupuncture therapy for cognitive impairment.

### Distribution of journals and highly cited articles

**Figure 4:**
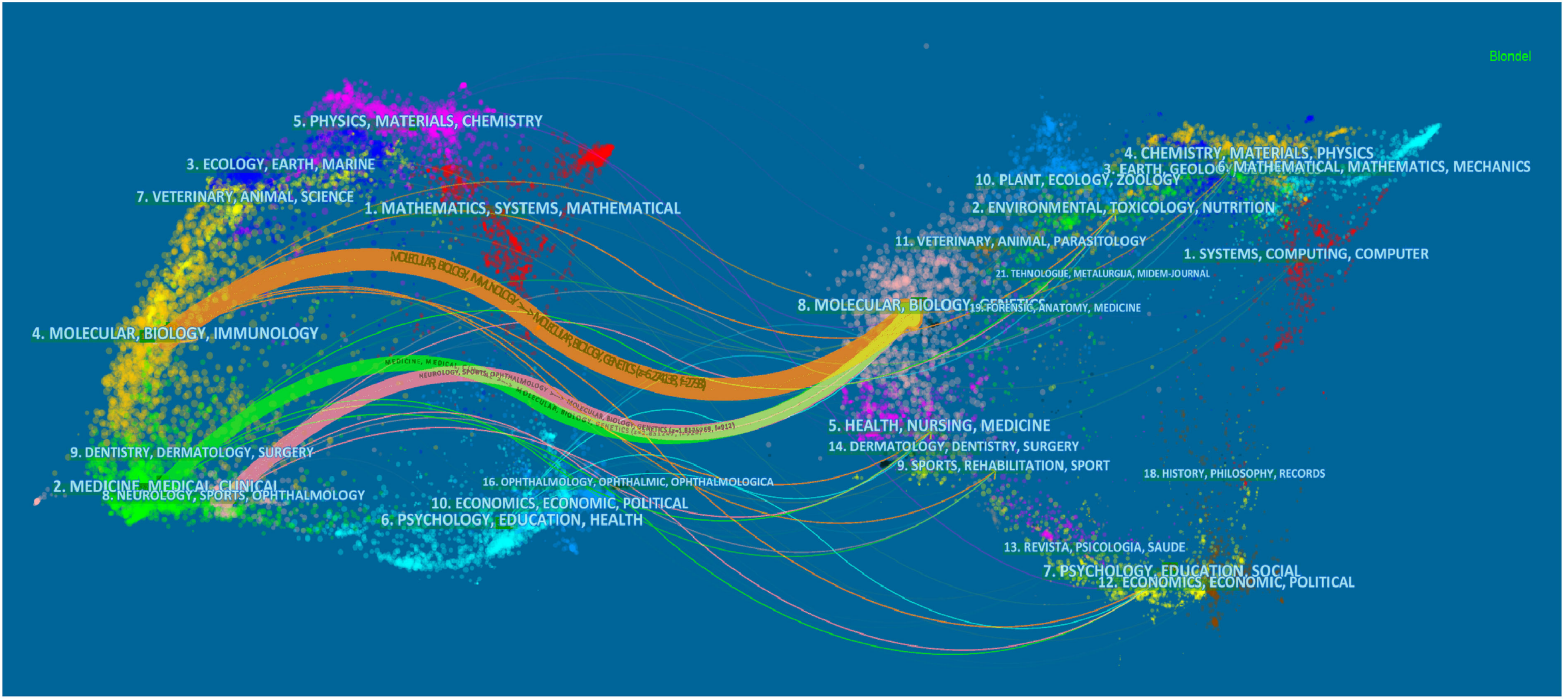
The dual-map overlay of journals. The left side correspond to the citation map and the right side represent the cited journal map. Molecular/biology/immunology journals represented by the yellow path, medicine/ medical/ clinical journals represented by the green path, and neurology/sports/ophthalmology journals represented by the pink path are cited in molecular/biology/genetics areas.

A total of 101 academic journals have published publications on acupuncture therapy for cognitive impairment. Table 1 lists the top 10 journals with a total of 137 articles. *Evidence-based Complementary and Alternative Medicine* published the most articles (*n* = 23), followed by *Trials* (*n* = 18) and *Neural Regeneration Research* (*n* = 14). Figure 4 displayed the dual-map overlay of journals (23), the left and right sides corresponded to the citation map and the cited journal map, respectively. These labels represented the disciplines covered by the journal. Lines on the map start from the left and end on the right, representing citation links. There were three citation paths: molecular/biology/immunology journals represented by the yellow path, medicine/medical/clinical journals represented by the green path, and neurology/sports/ophthalmology journals represented by the pink path are cited in molecular/biology/genetics areas. Table 2 shows the 10 most frequently cited articles.

**Table 1:** The top 10 journals that published articles.

**Rank**	**Journal**	**Country**	**Count**	**IF 2022**
1	Evidence-based Complementary and Alternative Medicine	England	23	2.650
2	Trials	England	18	2.728
3	Neural Regeneration Research	China	14	6.058
4	Acupuncture in Medicine	England	12	1.976
5	Medicine	United States	10	1.817
6	BMC Complementary and Alternative Medicine	England	9	4.782
7	Frontiers in Aging Neuroscience	Switzerland	9	5.702
8	Journal of Traditional Chinese Medicine	China	8	2.547
9	Neural Plasticity	United States	8	3.144
10	Neuroscience Letters	Netherlands	8	3.197

**Table 2:** Top 10 most cited articles.

**Rank**	**First/corresponding author**	**Title**	**Journal**	**Cited**	**Highlight**
1	Zhi-qun Wang/ Kun-cheng Li	Acupuncture Modulates Resting State Hippocampal Functional Connectivity in Alzheimer Disease	PLoS One	121	The study clarified that acupuncture at LR3 and LI4 could enhance the hippocampal connectivity in AD patients using fMRI
2	Yuan-yuan Feng/ Jie Tian	FMRI connectivity analysis of acupuncture effects on the whole brain network in mild cognitive impairment patients	Magnetic Resonance Imaging	75	The fMRI study showed that the correlations related to the temporal regions were enhanced in the poststimulus resting brain in MCI patients compared with healthy controls. Compared to superficial acupuncture at KI3, significantly increased correlations related to the temporal regions were found for the deep acupuncture condition.
3	Hai-yan Cheng/ Jing-Xian Han	Acupuncture improves cognitive deficits and regulates the brain cell proliferation of SAMP8 mice	Neuroscience Letters	73	The cognitive deficit of SAMP8 was revealed and significantly improved by “Yiqitiaoxue and Fubenpeiyuan” acupuncture (Sanjiao acupuncture). The experiment observed that the decreased cell proliferation in the dentate gyrus of SAMP8 was greatly enhanced by therapeutic acupuncture. a stream-like distribution of newly proliferated cells presented along the dorsum of alveus hippocampi, extending from the left ventricular to the corpus callosum.
4	Xiao-dong Feng/ Li-dian Chen	Electroacupuncture ameliorates cognitive impairment through inhibition of NF-kappa B-mediated neuronal cell apoptosis in cerebral ischemia-reperfusion injured rats	Molecular Medicine Reports	68	Electroacupuncture at GV20 and GV24 suppressed the I/R-induced activation of NF-κB signaling in ischemic cerebral tissues, which led to the inhibition of cerebral cell apoptosis. Furthermore, electroacupuncture markedly downregulated the expression of pro-apoptotic Bax and Fas, two critical downstream target genes of the NF-κB pathway.
5	Zhi-qun Wang/ Kun-cheng Li	Effect of Acupuncture in Mild Cognitive Impairment and Alzheimer Disease: A Functional MRI Study	PLoS One	66	To clarify the mechanisms of acupuncture at Tai LR3 and LI4 in treating MCI and AD patients by using fMRI.
6	Xu-ying Li/ Li-ze Xiong	Electroacupuncture decreases cognitive impairment and promotes neurogenesis in the APP/PS1 transgenic mice	BMC Complementary and Alternative Medicine	64	Electroacupuncture stimulation at GV20 significantly ameliorated the learning and memory deficits of APP/PS1 mice, decreased Aβ deposits, and increased brain-derived neurotrophic factor (BDNF) expression and neurogenesis in the hippocampus and cortex of EA-treated AD mice were detected.
7	Cun-Zhi Liu/ Jing-Xian Han	Acupuncture prevents cognitive deficits and oxidative stress in cerebral multi-infarction rats	Neuroscience Letters	63	suggesting that acupunctural prescription including CV17, CV12, CV6, ST36, and SP10 ameliorated oxidative injuries induced by cerebral multi-infarction by increasing the activities of superoxide dismutase (SOD) and glutathione peroxidase (GSH-Px) in the hippocampus.
8	Jian-chun Yu/ Jing-Xian Han	Acupuncture improved cognitive impairment caused by multi-infarct dementia in rats	Physiology & Behavior	62	The pattern of multi-infarct dementia in rats was made by injecting homogeneous emboli into the internal carotid artery. Acupunctural prescription including CV17, CV12, CV6, ST36, and SP10, the present results suggested that acupuncture exerted a protective effect on cognitive impairment caused by cerebral multi-infarction in rats, and acupuncture has a specificity of cure.
9	Bombi Lee/ Hyejung Lee	Acupuncture stimulation improves scopolamine-induced cognitive impairment *via* activation of cholinergic system and regulation of BDNF and CREB expressions in rats	BMC Complementary and Alternative Medicine	57	This study aimed to examine whether acupuncture stimulation at GV20 improves memory defects caused by scopolamine (SCO) administration in rats. The result showed that acupuncture significantly alleviated memory-associated decreases in the levels of choline acetyltransferase (ChAT), BDNF, and cAMP-response element-binding protein (CREB) proteins in the hippocampus. Moreover, acupuncture restored the expression of choline transporter 1 (CHT1), vesicular acetylcholine transporter (VAChT), BDNF, and CREB mRNA in the hippocampus.
10	Li-Chan Lin	Using Acupressure and Montessori-Based Activities to Decrease Agitation for Residents with Dementia: A Cross-Over Trial	Journal of the American Geriatrics Society	55	A double-blinded, randomized cross-over design was used to evaluate the effectiveness of acupressure and Montessori-based activities in decreasing the agitated behaviors of residents with dementia. Results mainly demonstrated that the acupressure and Montessori-based activities groups saw a significant decrease in agitated behaviors, aggressive behaviors, and physically nonaggressive behaviors than the presence group.

### Distribution of countries and institutes

**Figure 5:**
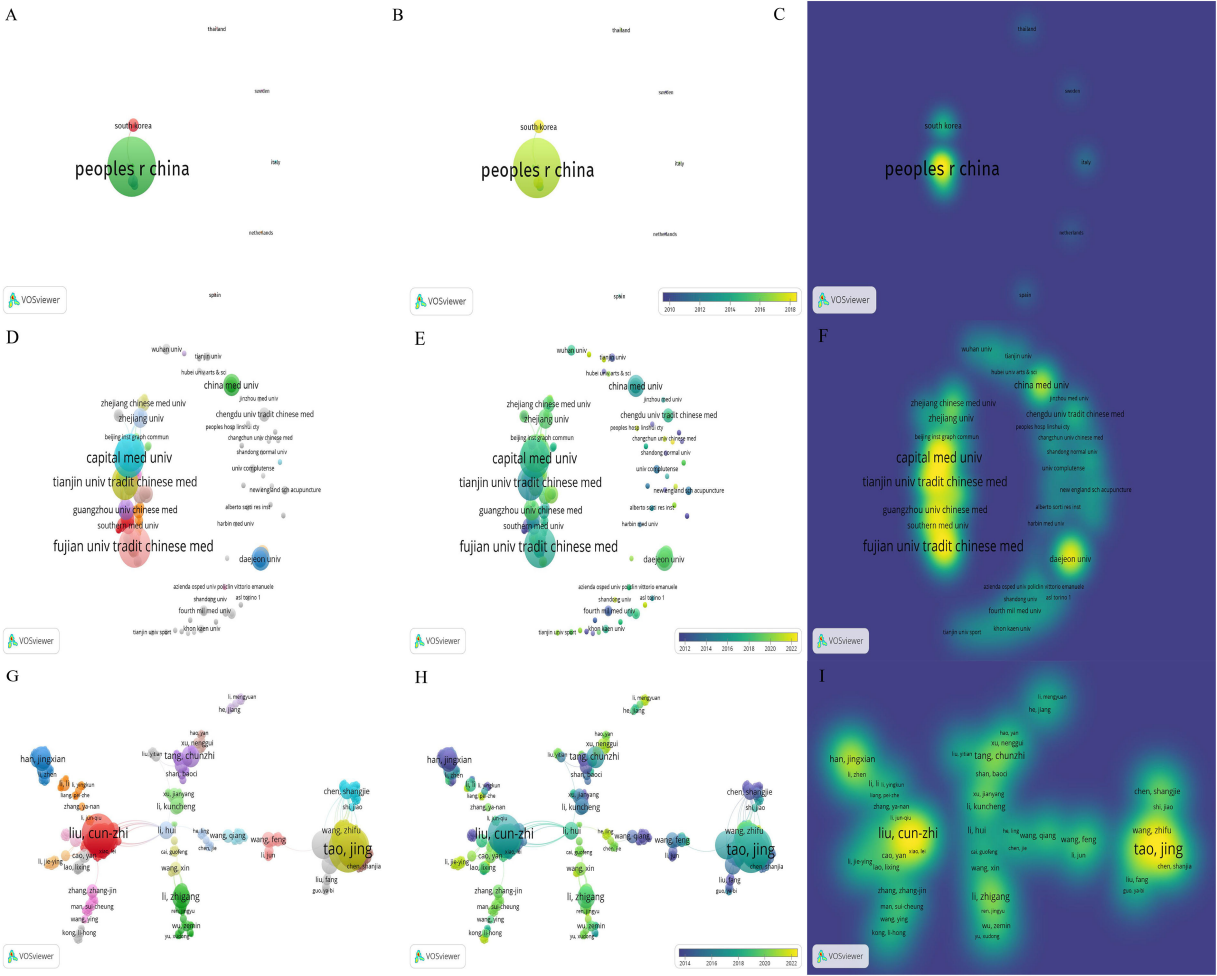
The network map of countries/regions, institutes, and active authors. **(A)** The Network Visualization map of countries/regions, **(B)** the Overlay Visualization map of countries/regions, and **(C)** the Density Visualization map of countries/regions. **(D)** The Network Visualization map of institutes, **(E)** the Overlay Visualization map of institutes, and **(F)** the Density Visualization map of institutes. **(G)** The Network Visualization map of active authors, **(H)** the Overlay Visualization map of active authors, and **(I)** the Density Visualization map of active authors.

A total of 19 countries/regions have published research publications on acupuncture therapy for cognitive impairment, and extensive cooperation between countries/regions has been observed (Figure 5). Table 3 lists the top 10 countries/regions in the number of publications, of which China is the most, followed by South Korea, the United States, and England. At present, the research on acupuncture treatment of CI is mainly concentrated in China and South Korea. With the increase of international exchanges in recent years, the United States and England are gradually increasing their participation in research, and the multi-country cooperation model is gradually being carried out.

**Table 3:** Top 10 countries/region and institutions in the number of publications.

**Rank**	**Country/region**	**Count**	**Institute**	**Count**
1	China	217	Fujian University of Traditional Chinese Medicine	40
2	South Korea	26	Capital Medical University	34
3	the United States	24	Beijing University of Chinese Medicine	31
4	England	5	Tianjin University of Traditional Chinese Medicine	26
5	Italy	4	Guangzhou University of Chinese Medicine	17
6	Australia	2	Korea Institute of Oriental Medicine	14
7	Canada	2	China Medical University (Taiwan)	13
8	Germany	2	Shanghai University of Traditional Chinese Medicine	11
9	Norway	2	Southern Medical University	10
10	Spain	2	Chinese Academy of Sciences	9

In total, 334 institutes participated in acupuncture research (Figure 5). Table 3 lists the top 10 institutes in terms of publications. It should be noted that we have merged different names of the same institutes, such as Fujian University of TCM merged into Fujian University of Traditional Chinese Medicine, Guangzhou University of Traditional Chinese Medicine merged into Guangzhou University of Chinese Medicine. Statistics showed that the top 10 institutes account for 73.48% of total publications, among which Fujian University of Traditional Chinese Medicine has the largest publications, followed by Capital Medical University and Beijing University of Chinese Medicine.

### Analysis of citations and H-index

**Figure 6:**
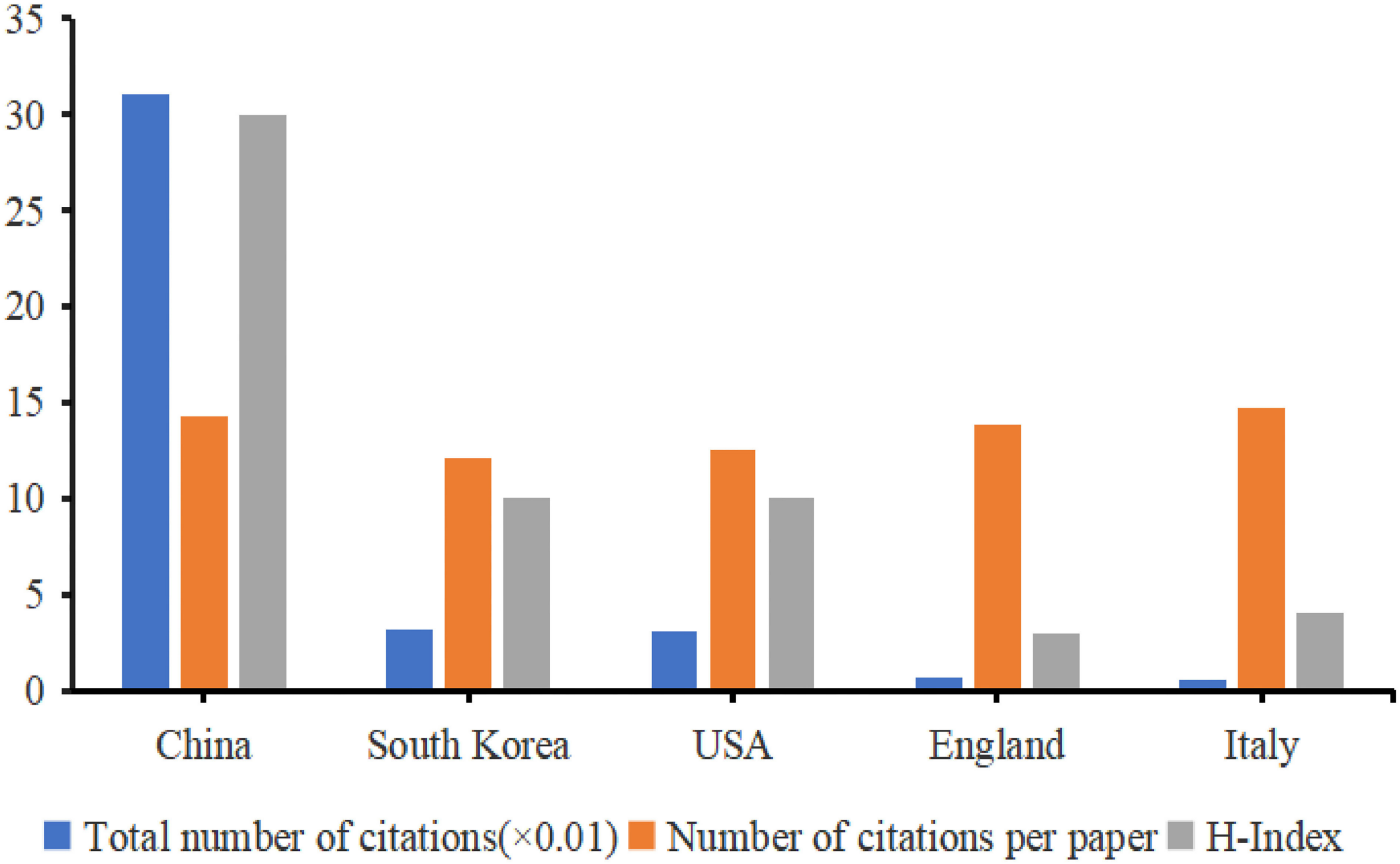
The distribution of citation (× 0.01), number of citations per article, and H-index in the top 5 countries. According to the number of citations and H-index, the histogram intuitively shows the top 5 countries, among which Chinese scholars have done the most research in this field and have the highest influence.

China ranked first among the top five productive countries in terms of the total number of citations and H-index, followed by South Korea, the United States, England, and Italy (Figure 6). All countries have not contributed to the ESI top articles. High-impact research achievements are still needed in this field.

### Analysis of authors

Over 1,210 authors contributed to acupuncture research. The co-occurrence map of authors is shown in Figure 5. Table 4 lists the top 10 authors in the number of publications. Li-Dian Chen and Cun-Zhi Liu (26 publications) were both ranked first, followed by Jing Tao (25 publications) and Jia Huang (19 publications).

**Table 4:** The top 10 authors, co-cited authors, and keywords.

**Rank**	**Author**	**Count**	**Co-cited Author**	**Count**	**keyword**	**Count**
1	Li-Dian Chen	26	Ronald C Petersen	56	Alzheimer's disease	102
2	Cun-Zhi Liu	26	Zhi-Qun Wang	53	mild cognitive impairment	97
3	Jing Tao	25	Jian-Chun Yu	46	acupuncture	57
4	Jia Huang	19	Li-Jun Bai	42	vascular dementia	45
5	Wei-Lin Liu	15	Lan Zhao	38	dementia	43
6	Jing-Wen Yang	15	Yuan-yuan Feng	34	memory	34
7	Jing-Xian Han	12	Ru-Hui Lin	33	brain	33
8	Ru-Hui Lin	12	Jing Zhou	33	stroke	30
9	Xue-Rui Wang	12	Cun-Zhi Liu	29	activation	30
10	Jian-Chun Yu	12	Guang-Xia Shi	29	expression	27

**Figure 7:**
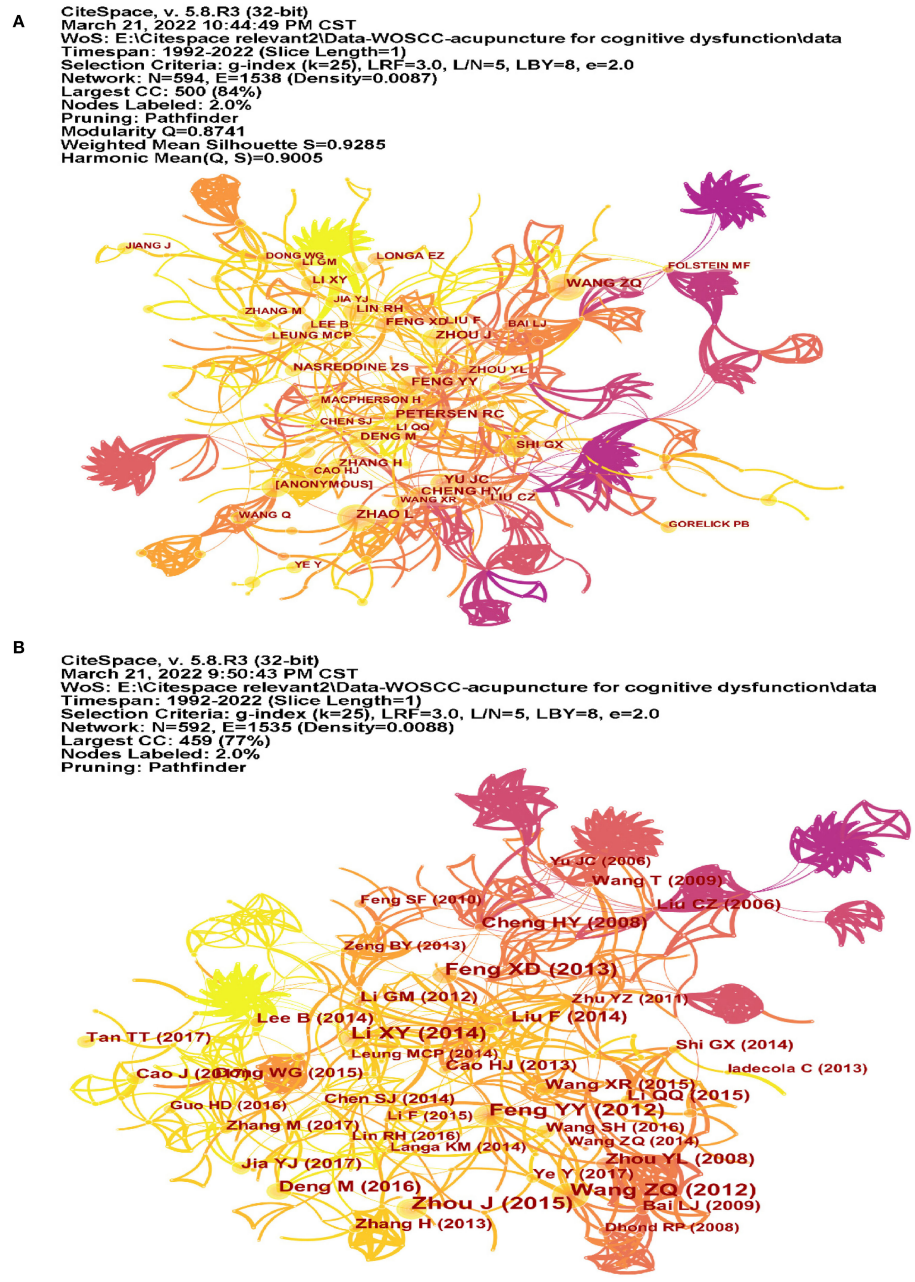
The co-citation map of authors and references. **(A)** The co-citation map of authors and **(B)** the co-citation map of authors references.

**Figure 8:**
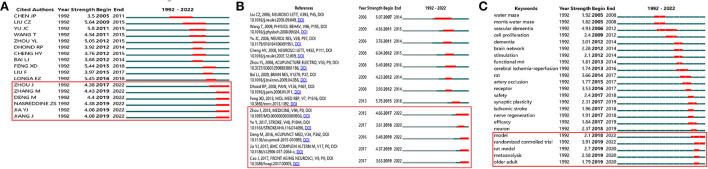
The co-cited authors, co-cited references, and keyword with the strongest citation bursts. **(A)** The co-cited authors with the strongest citation bursts, **(B)** the co-cited references with the strongest citation bursts, and **(C)** the keyword with the strongest citation bursts. Among them, the co-cited authors, co-cited references, and keywords in the red box are the most obvious in the past 3 years, which may be the latest research hotspots.

The co-citation of authors was analyzed by CiteSpace (Figure 7A). Among the top 10 co-cited authors (Table 4), Ronald C Petersen (56 co-citations) ranked first, followed by Zhi-Qun Wang (53 co-citations), and Jian-Chun Yu (46 co-citations). The top 17 authors with the strongest citation bursts are listed in Figure 8A, and the beginning to the end of each burst interval is indicated by a red line. Jing Zhou (strength 4.38, 2017–2022), Meng Zhang (strength 4.43, 2019–2022), Min Deng (strength 4.40, 2019–2022), Nasreddine ZS (strength 4.38, 2017–2022), Yang-Juan Jia (strength 4.08, 2019–2022), and Jing Jiang (strength 4.08, 2019–2022) have been cited in recent 3 years, indicating that the authors have been active in this field in recent years. New research has been published in the study of cognitive impairment.

### Analysis of co-cited references

**Figure 9:**
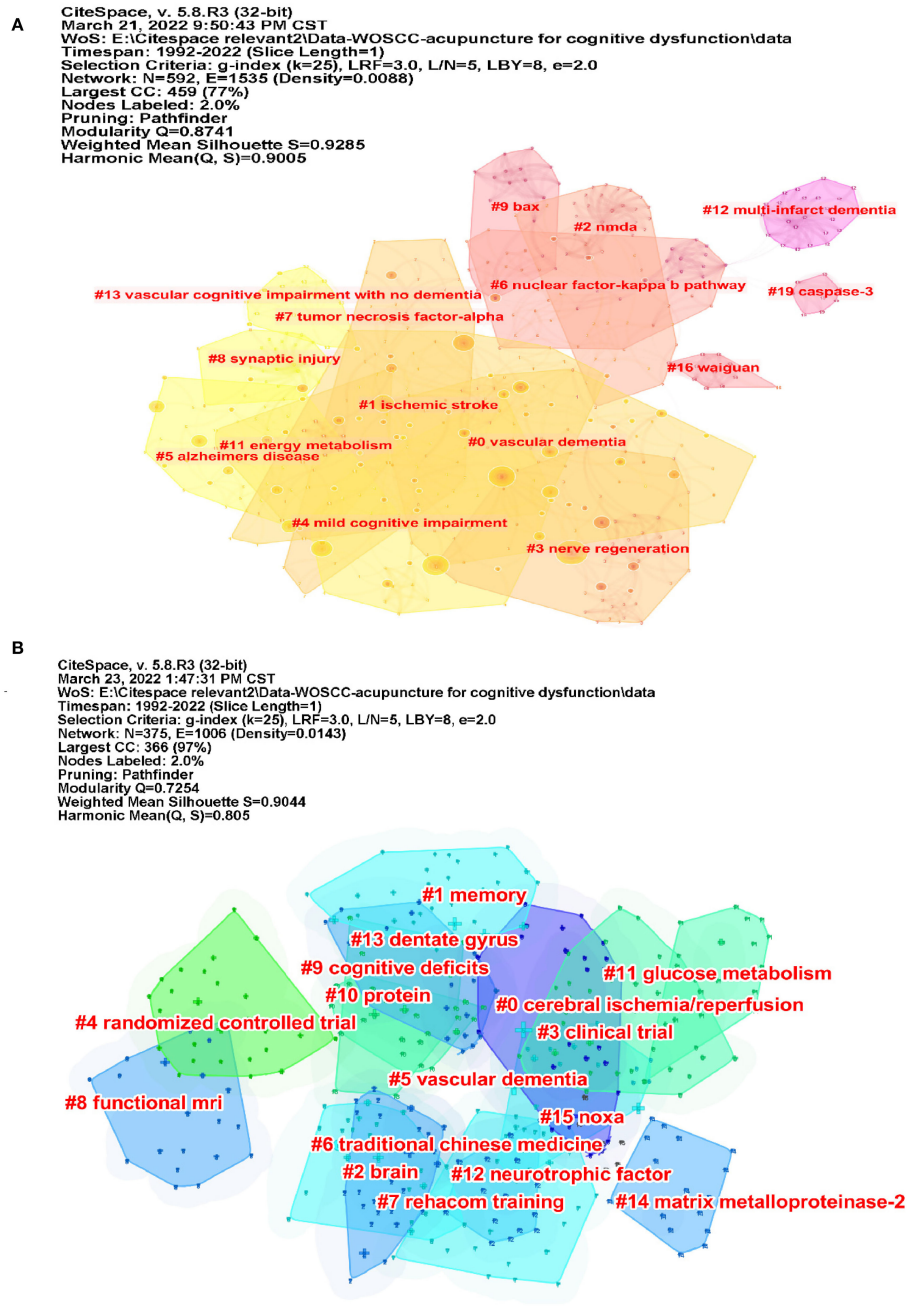
The co-cited reference and keyword clusters map for publications. **(A)** The co-cited reference clusters map for publications and **(B)** the keyword clusters map for publications. In clustering, the same elements would be aggregated together, each cluster existed independently, and the cluster label is a name for each cluster diagram.

We used CiteSpace to analyze the co-citation of references (Figure 7B). Table 5 lists the top 10 co-cited references. The co-cited reference clusters are shown in Figure 9A. The network contained 592 nodes and 1,535 links. The Modularity Q was 0.8741 (>0.5), meaning that the clusters of networks were reasonable, and the Mean Silhouette S was 0.9285 (>0.5), indicating that the homogeneity of clusters were acceptable (20). In this network, more important clustering labels were listed in 20 clusters: #0 vascular dementia, #1 ischemic stroke, #2 NMDA, #3 nerve regeneration, #4 mild cognitive impairment, #5 Alzheimer's disease, #6 nuclear factor-kappa b pathway, #7 tumor necrosis factor-alpha, #8 synaptic injury, #9 Bax, #11 energy metabolism, #12 multi-infarct dementia, #13 vascular cognitive impairment with no dementia, #16 waiguan (SJ5), and #19 Caspase-3. The top 13 co-cited references with the strongest citation bursts are listed in Figure 8B.

**Table 5:** The top 10 co-cited references.

**Rank**	**Co-cited reference**	**Title**	**Count**
1	Zhou J, 2015, MEDICINE, V94, P0, DOI 10.1097/MD.0000000000000933	The effectiveness and safety of acupuncture for patients with Alzheimer disease: a systematic review and meta-analysis of randomized controlled trials	24
2	Wang ZQ, 2012, PLOS ONE, V7, P0, DOI 10.1371/journal.pone.0042730	Effect of acupuncture in mild cognitive impairment and Alzheimer disease: a functional MRI study	23
3	Feng YY, 2012, MAGN RESON IMAGING, V30, P672, DOI 10.1016/j.mri.2012.01.003	FMRI connectivity analysis of acupuncture effects on the whole brain network in mild cognitive impairment patients	22
4	Feng XD, 2013, MOL MED REP, V7, P1516, DOI 10.3892/mmr.2013.1392	Electroacupuncture ameliorates cognitive impairment through inhibition of NF-κB-mediated neuronal cell apoptosis in cerebral ischemia-reperfusion injured rats	20
5	Li XY, 2014, BMC COMPLEM ALTERN M, V14, P0, DOI 10.1186/1472-6882-14-37	Electroacupuncture decreases cognitive impairment and promotes neurogenesis in the APP/PS1 transgenic mice	19
6	Deng M, 2016, ACUPUNCT MED, V34, P342, DOI 10.1136/acupmed-2015-010989	Acupuncture for amnestic mild cognitive impairment: a meta-analysis of randomized controlled trials	15
7	Liu F, 2014, J ALTERN COMPLEM MED, V20, P535, DOI 10.1089/acm.2013.0364	A meta-analysis of acupuncture use in the treatment of cognitive impairment after stroke	15
8	Zhou YL, 2008, ACUPUNCTURE ELECTRO, V33, P9, DOI 10.3727/036012908803861186	Effect of acupuncture given at the HT7, ST36, ST40 and KI3 acupoints on various parts of the brains of Alzheimer's disease patients	14
9	Cheng HY, 2008, NEUROSCI LETT, V432, P111, DOI 10.1016/j.neulet.2007.12.009	Acupuncture improves cognitive deficits and regulates the brain cell proliferation of SAMP8 mice	14
10	Li QQ, 2015, PHYSIOL BEHAV, V139, P482, DOI 10.1016/j.physbeh.2014.12.001	Hippocampal cAMP/PKA/CREB is required for neuroprotective effect of acupuncture	13

### Analysis of keywords

**Figure 10:**
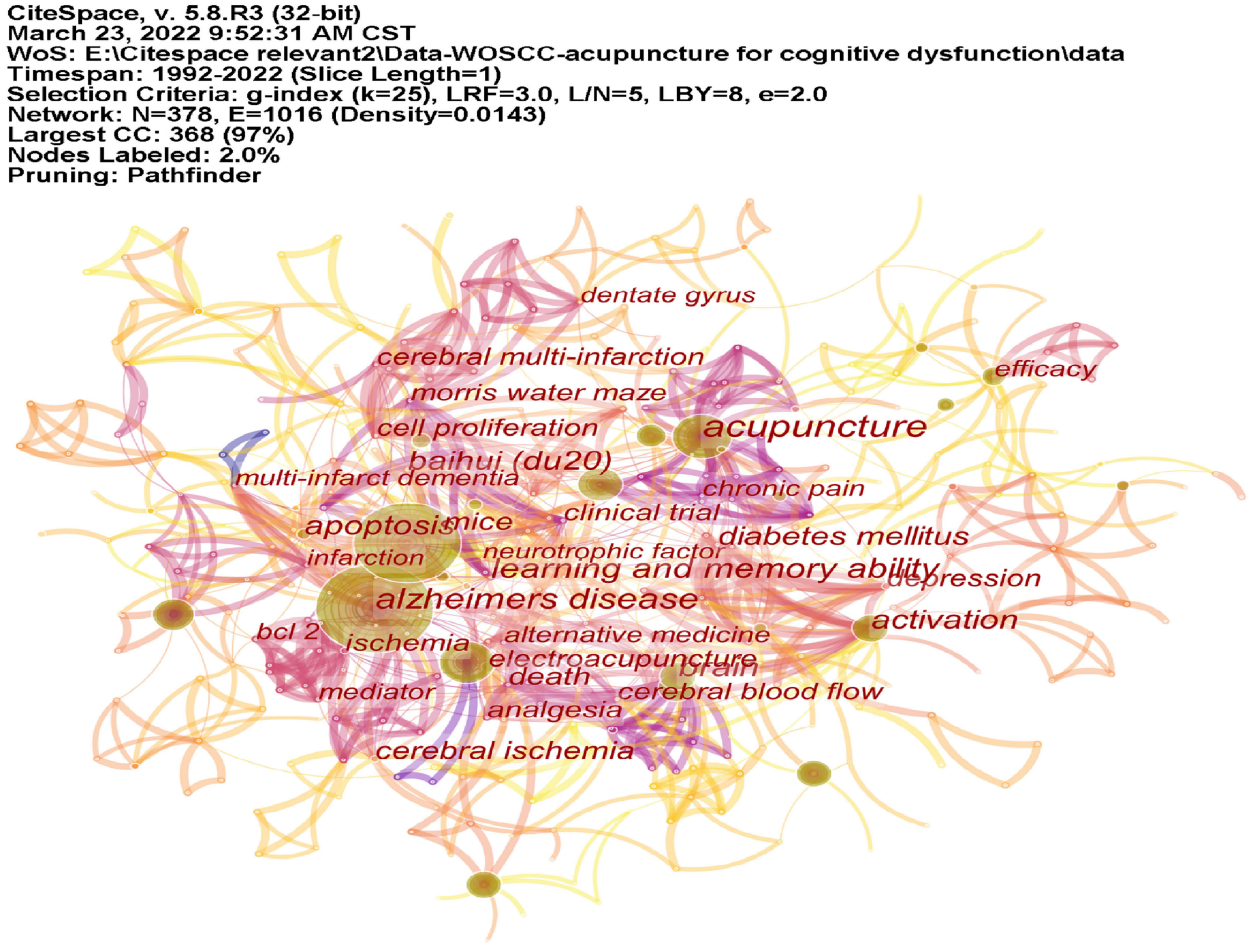
The co-occurrence map of keywords. CiteSpace was used to analyze the co-occurrence of keywords. The figure shows that the keywords, such as Alzheimer's disease (AD), acupuncture, electroacupuncture, brain, activation, and Baihui occupied the main position.

CiteSpace was used to analyze the co-occurrence of keywords (Figure 10). Table 4 lists the top 10 keywords. Co-cited keyword clusters network contained 375 nodes and 1,006 links. the Modularity Q was 0.7254, and the Mean Silhouette S was 0.9044 (>0.5) (Figure 9B). There were 16 clustering labels listed: #0 cerebral ischemia/reperfusion, #1 memory, #2 brain, #3 clinical trial, #4 randomized controlled trial, #5 vascular dementia, #6 traditional Chinese medicine, #7 rehacom training, #8 functional MRI, #9 cognitive deficits, #10 protein, #11 glucose metabolism, #12 neurotrophic factor, #13 dentate gyrus, #14 matrix metalloproteinase-2 (MMP-2), and #15 Noxa. The top 23 co-cited keywords with the strongest citation bursts are listed in Figure 8C. As shown in the figure, the part selected by the red square represents the highly cited keywords in recent years, which are mainly reflected in randomized controlled trials, meta-analysis, and the construction of animal models.

## Discussion

### General information

The number of publications and citations has shown a rapid upward trend every year, indicating that acupuncture therapy for MCI research is still attracting attention. Among the top 10 contributing countries, China and South Korea accounted for the largest proportion (87.10%). Research institutions are also represented by traditional Chinese medicine colleges, which reflects the high acceptance of acupuncture in Asia. In terms of the distribution of authors, Li-Dian Chen ranked first in 26 publications. According to Price's Law (24), the minimum number of publications for core authors is *N* = 0.749$\sqrt[{}]{M\max}$ (*M*max is the publications of the most prolific authors), calculated at *N* ≈ 4. In terms of the number of articles published, 89 authors have published more than 4 articles, accounting for 7.36% (< 50%), indicating that the core author team in this research field has not yet been formed. At present, scholars who study acupuncture for CI are mainly concentrated in Asia and relatively few in Europe and the United States, which may have a certain relationship with the culture they come into contact with. It is necessary to popularize acupuncture and let more people know about it. At the same time, strengthening the cooperation and exchanges between scholars from various countries will help to further explore the effective mechanism of acupuncture treatment. Perhaps this will not only be limited to the study of improving cognitive function.

### Research hotspots and frontiers

#### RCTs of acupuncture therapy for MCI

Earlier identification and intervention of mild cognitive impairment are necessary to delay the progression of the disease to dementia. The results of the meta-analysis based on RCTs suggested that acupuncture is effective in the treatment of patients with MCI and can improve their activity on a daily living scale, the mini-mental state examination (MMSE), and Montreal cognitive assessment scores (MoCA) (25). As mentioned in the preface, the lack of high-quality RCT articles is the main reason leading to the medium-low quality evidence of acupuncture treatment CI. There is still a need for standardized reports on acupuncture research.

#### Animal models focus on AD and VaD

Alzheimer's disease models involved in this study include the rapid aging models, transgenic models, and Aβ injection models. Senescence-Accelerated Mice include two strains, SAM-P and SAM-R (26, 27). SAM-P exhibits rapid aging, AD-specific Aβ aggregation, and hyperphosphorylation of Tau protein in pathology. SAM-P has 9 sub-strains, among which SAMP8 is an ideal animal model for aging-related learning and memory deficits that are similar to the clinical manifestations of patients with AD (28). SAMR1 mice retain normal aging characteristics and are often used as controls. The transgenic animal models involved in this study are amyloid precursor protein/Presenilin 1 (APP/PS1) mice and five familial mutations (5XFAD) mice. APPSwe/PS1 (B6C3-Tg (APPswe, PSEN1dE9) 85Dbo/J) double transgenic mice model. APPSwe is the Swedish mutation of the amyloid precursor protein, whereas PS1 is the mutant form of human presenilin 1 (29). 5XFAD mice overexpress human amyloid precursor protein (APP) and presenilin-1 (PS1) mutants, namely, the Swedish (K670 N and M671 L), Florida (I716V), and London (V717I) mutations in APP and the PS1 mutations M146 L and L286 V (30). The above two kinds of transgenic animals can specifically produce excessive APP, which affects the accumulation of Aβ, and finally forms senile plaques (SP). Aβ injection models are used to inject Aβ polypeptide fragments of different lengths into specific brain regions, such as Aβ1-40 (31) and Aβ1-42 (32). It is worth mentioning that all the animal studies on the acupuncture treatment of AD seem to be directed at the regulatory mechanism of Aβ. At present, there are few studies on tauopathies, which we believe can be further studied in the future.

For VaD models, the permanent, bilateral common carotid artery occlusion (2-VO) is most commonly used in this study (33). By ligating the common carotid artery, a chronic cerebral hypoperfusion state is created, thereby causing ischemia and hypoxia in brain tissue, especially the hippocampus and cortex. However, this modeling method has a high lethality rate, and the modified version of 2-VO can improve the survival rate.

#### Various putative mechanisms of acupuncture in the treatment of AD and VaD

The putative mechanism of acupuncture in the treatment of AD and VaD mainly focuses on the repair of neuroinflammation, regulating autophagy and apoptosis, and improving energy metabolism. Tables 6, 7 lists the different putative mechanisms of acupuncture for AD (34–70) and VaD (71–85). It is worth noting that Baihui (GV20, also called DU20) occupies the most acupuncture points, prompting its importance in acupoint selection. Furthermore, the selection of acupuncture points is based on the theory of Chinese medicine, and most of the points are not at least one. The final result makes it difficult to determine whether it is the effect of single point stimulation or superposition of combination. In the experimental grouping, in addition to setting up the sham group, the setting of a single acupoint or combination needs further consideration.

**Table 6:** The putative mechanisms of acupuncture on different Alzheimer's disease animal models.

**Model**	**Acupuncture point**	**Mechanism**
SAMP8 mice	CV17, CV12, CV6, ST36, SP10	Regulates brain cell proliferation (34). Reduced neuron loss in hippocampal regions CA3 and DG (35). upregulated the expression of bFGF, EGF, and BDNF (36). improving synaptophysin mRNA and protein levels (37). promoting Hsp84 and Hsp86 expression (38). accelerates synaptophysin production (39). Down-regulating PI3K/PDK1/nPKC/Rac1 signaling pathway (40).
	GV14, BL23	increased the levels of p-AMPK (41). upregulated the expression of SIRT1 and PGC-1α (42). downregulation of BACE1(43).
	GV20, GV29	increased CBF in the prefrontal lobe and hippocampus (44). by balancing the gut microbiota (45). enhanced paravascular influx in the glymphatic system inhibited the reactivity of astrocytes and improved AQP4 polarity (46).
	GV20, GV29, GV26	improved the level of glucose metabolism (47).
	GV20, BL23	inhibited the AMPK/eEF2K/eEF2 signaling pathway (48).
	GV20, ST36	downregulated NLRP3/caspase-1 pathway (49).
	GV20, BL23, KI3	inhibited activation of astrocytes and microglia and decreased expression of pro-inflammatory cytokines, TNF-α, and IL-17 (50).
APP/PS1 mice	GV20	up-regulated the expression of BDNF (51, 52). Induced phosphorylated AMPK and AKT inhibited the phosphorylation level of the mammalian target of mTOR (53). suppressed GFAP and NDRG2 upregulation (54). increased the expression levels of BDNF and proBDNF, p-TrkB was upregulated, and p75NTR was decreased (55).
	GV20, GV29, GV26	enhancing glucose metabolism (56, 57). downregulated of BACE1, p-PKA protein (58). inhibited JNK signaling pathway (59). induced AKT (Ser473) and GSK3β (Ser9) phosphorylation, inhibited the phosphorylation of Tau (Ser199 and Ser202) proteins (60).
	GV20, BL23	reduced the expressions of BACE1, and increased the expression of IDE protein (61)^.^
	GV20, GV24	activated AMPK to enhance the process of Aerobic glycolysis (AG), and enhanced glucose metabolism (62).
5x FAD	KI3	inhibition of neuroinflammation and increased glucose metabolism (63). upregulation of synaptophysin and postsynaptic density-95 protein (64).
	GV24, GB13	activated TFEB *via* inhibiting the AKT-MAPK1-MTORC1 pathway (65).
	GV20, GV24	activating the medial septal and vertical limb of the diagonal band and dentate gyrus (MS/VDB-DG) cholinergic neural circuit (66).
injecting Aβ_1 − 40_ Rat Model	GV20, BL23	activation of PPAR-γ and inhibition of p-p38MAPK expression (67). upregulated the expression of Bcl-2 and downregulated the expression of Bax, downregulated the level of Notch1 and Hes1 mRNA in the hippocampus (68).
injecting Aβ_1 − 42_ Rat Model	GV20, BL23	downregulated the expression of GSK-3β (69).
	GV29, LI20	the activation level of PI3K/AKT signaling and the phosphorylation inactivation of GSK-3β (70).

Dentate Gyrus (DG), basic fibroblast growth factor (bFGF), epidermal growth factor (EGF), brain-derived neurotrophic factor (BDNF), heat shock protein (Hsp), Phosphatidylinositol 3 Kinase (PI3K), Phosphoinositol-Dependent Kinase 1 (PDK1), Novel Protein Kinase C (nPKC), Sirtuin 1 (SIRT1), proliferator-activated receptor-γ-co-activator-1α (PGC-1α), Beta-secretase 1 (BACE1), Cerebral blood flow (CBF), aquaporin-4 (AQP4), AMP-activated protein kinase (AMPK), eukaryotic elongation factor-2 kinase (eEF2K), eukaryotic elongation factor-2 (eEF2), Nod-like receptor family pyrin domain containing 3 (NLRP3), tumor necrosis factor-α (TNF-α), interleukin-17 (IL-17), thymoma viral proto-oncogene (AKT), glial fibrillary acidic protein (GFAP), N-myc downstream-regulated gene 2 (NDRG2), c-Jun N-terminal kinase(JNK), insulin degrading enzyme (IDE), mitogen-activated protein kinase 1 (MAPK1), mechanistic target of rapamycin kinase complex 1 (MTORC1), glycogen synthase kinase-3β (GSK-3β).

**Table 7:** Mechanisms of acupuncture on different vascular dementia animal models.

**Model**	**Acupuncture point**	**Mechanism**
using the permanent, bilateral common carotid artery occlusion (2VO)	GV20, ST36	Up-regulate the protein and mRNA levels of Nrf2 and its target genes HO-1 and NQO1 (71). up-regulated the expressions of Trx-1 and TrxR-1 and inhibited the activation of the ASK1/JNK/ p38 pathway (72). downregulated the expression of TXNIP, NLRP3, caspase-1, and IL-1β (73). downregulated the expression of TLR4, accompanied by a decrease in microRNA-93 and MyD88/ NF-κB signaling pathway activation. activated of D1/ D5 receptors (74). activated of D1/D5 receptors (75). increased the expression of Hippocampal mitochondrial respiratory complex enzymes (complex I, II, IV) activities and cytochrome c oxidase IV expression (76). increased CBF attenuated the loss of myelin basic protein and microglial accumulation associated with IL-1β and IL-6 production (77). decreased ROS production and improved LTP (78).
	GV20, GV14, BL23	up-regulated expression of mTOR and eIF4E (79). inhibited expression of Noxa and caspase-3 expression (80). blocked expression of p53 and Noxa (81).
	GV20, GV14	promoted synaptophysin expression (82).
four-vessel occlusion (4-VO)	GV20, CV17, BL17, CV6, SP6	inhibited the protein and mRNA expressions of TLR4 and MyD88 in the hippocampus of rats, and reduced the expressions of serum IL-6 and TNF-α (83).
*Via* bilateral middle cerebral artery occlusion	GV20, GV14, BL23	up-regulated expression of p70 ribosomal protein S6 kinase and ribosomal protein S6 (84).
with 3% microemboli saline suspension	ST36	increased the pyramidal neuron number (85).

Nuclear factor E2 related factor 2 (Nrf2), thioredoxin-1 (Trx-1), thioredoxin reductase-1 (TrxR-1), apoptosis signal-regulating kinase 1 (ASK1), c-Jun N-terminal kinase (JNK), Thioredoxin-interacting protein (TXNIP), interleukin-1β (IL-1β), Toll-like receptors 4 (TLR4), myeloid differentiation factor 88 (MyD88), nuclear factor-kappa B (NF-κB), reactive oxygen species (ROS), long-term potentiation (LTP), rapamycin (mTOR), eukaryotic translation initiation factor 4E (eIF4E), tumor necrosis factor-α (TNF-α), interleukin-6 (IL-6).

#### Functional magnetic resonance imaging is one of the important tools to research cognitive function

Functional magnetic resonance imaging is a non-invasive neuroimaging method with a high spatial resolution to study brain function. Its method of detecting functional connectivity of various brain regions in resting or task states may objectively identify cognitive impairment. Acupuncture in the cognitive impairment group compared with healthy controls to observe whether cognitive-related brain areas (such as dentate gyrus, frontotemporal lobe, and frontal lobe) are activated (86–88).

### Limitations

This study also has some disadvantages: first, our study was conducted on 20 March 2022, and included all articles up to 01 March 2022, but since the WoSCC database is still open to relevant documents in 2022, this section is omitted. Second, since each published article was limited to 3–10 keywords, some core words in these articles were not included in the bibliometric analysis, so the analysis results may also be affected by incomplete keyword extraction. Finally, as the search was limited to journals indexed in the WoSCC database, some articles not included in the WOSCC database were left out. Other bibliometric studies have also reported these limitations (89–91).

## Conclusion

We analyzed the research progress of acupuncture in the treatment of CI through the visualization analysis, and this study shows the current achievements of acupuncture in the treatment of CI and the possible directions of further research in the future, which will be helpful for clinicians and researchers. The results suggest that diseases focus on MCI, AD, and VaD. Pathologically, the detection of Aβ and APP is the main concern. Tauopathies with hyperphosphorylation of Tau protein as the main lesions also need to be paid attention to. In addition, fMRI is one of the means to elucidate the mechanism of treatment. At the same time, the complexity of acupoint selection makes it difficult to explain the specific pathway mechanism of acupuncture treatment of diseases. We think it is necessary to select a single point, and we found that Baihui (GV20) is the most commonly used, so we suggest that researchers can further elaborate on the mechanism of treating CI with GV20 acupuncture. Finally, the implementation of high-quality multicenter randomized controlled trials requires increased collaboration among experts from multiple fields and countries.

## Data availability statement

The original contributions presented in the study are included in the article/Supplementary material, further inquiries can be directed to the corresponding author/s.

## Author contributions

RZ and LX conceived and designed the experiments, authored, and reviewed drafts of the paper. RZ wrote the original draft. YY, RZ, WX, and LX analyzed the data. LX reviewed and edited the final draft. HWa, YY, RZ, LX, and WX performed the experiments and approved the final draft. HWa, YY, and WX prepared figures and/or tables. All authors contributed to the article and approved the submitted version.

## Conflict of interest

The authors declare that the research was conducted in the absence of any commercial or financial relationships that could be construed as a potential conflict of interest.

## Publisher's note

All claims expressed in this article are solely those of the authors and do not necessarily represent those of their affiliated organizations, or those of the publisher, the editors and the reviewers. Any product that may be evaluated in this article, or claim that may be made by its manufacturer, is not guaranteed or endorsed by the publisher.

## References

[1] Sachdev PS Blacker D Blazer DG Ganguli M Jeste DV Paulsen JS . Classifying neurocognitive disorders: the DSM-5 approach. Nat Rev Neurol. (2014) 10:634–42. 10.1038/nrneurol.2014.181 25266297

[2] Reed GM First MB Kogan CS Hyman SE Gureje O Gaebel W . Innovations and changes in the ICD-11 classification of mental, behavioural and neurodevelopmental disorders. World Psychiatry. (2019) 18:3–19. 10.1002/wps.20611 30600616 PMC6313247

[3] Petersen RC. Mild cognitive impairment as a diagnostic entity. J Intern Med. (2004) 256:183–94. 10.1111/j.1365-2796.2004.01388.x 15324362

[4] Langa KM Levine DA. The diagnosis and management of mild cognitive impairment: a clinical review. JAMA. (2014) 312:2551–61. 10.1001/jama.2014.13806 25514304 PMC4269302

[5] Gauthier S Reisberg B Zaudig M Petersen RC Ritchie K Broich K . Mild cognitive impairment. Lancet. (2006) 367:1262–70. 10.1016/S0140-6736(06)68542-5 16631882

[6] Ritchie K LovestONE S. The dementias. Lancet. (2002) 360:1759–66. 10.1016/S0140-6736(02)11667-9 12480441

[7] Association As. (2021 Alzheimer's disease facts and figures. Alzheimers Dement. (2021) 17:327–406. 10.1002/alz.12328 33756057

[8] Jia B Liu Z Min B Wang Z Zhou A Li Y . The effects of acupuncture at real or sham acupoints on the intrinsic brain activity in mild cognitive impairment patients. Evid Based Complement Alternat Med. (2015) 2015:529675. 10.1155/2015/529675 26064166 PMC4433670

[9] Chen S Xu M Li H Liang J Yin L Liu X . Acupuncture at the Taixi (KI3) acupoint activates cerebral neurons in elderly patients with mild cognitive impairment. Neural Regen Res. (2014) 9:1163–8. 10.4103/1673-5374.135319 25206776 PMC4146092

[10] Jia Y Zhang X Yu J Han J Yu T Shi J . Acupuncture for patients with mild to moderate Alzheimer's disease: a randomized controlled trial. BMC Complement Altern Med. (2017) 17:556. 10.1186/s12906-017-2064-x 29284465 PMC5747102

[11] Wang S Yang H Zhang J Zhang B Liu T Gan L . Efficacy and safety assessment of acupuncture and nimodipine to treat mild cognitive impairment after cerebral infarction: a randomized controlled trial. BMC Complement Altern Med. (2016) 16:361. 10.1186/s12906-016-1337-0 27623621 PMC5022140

[12] Feng X Yang S Liu J Huang J Peng J Lin J . Electroacupuncture ameliorates cognitive impairment through inhibition of NF-κB-mediated neuronal cell apoptosis in cerebral ischemia-reperfusion injured rats. Mol Med Rep. (2013) 7:1516–22. 10.3892/mmr.2013.1392 23525450

[13] Yang Y Hu S Lin H He J Tang C. Electroacupuncture at GV24 and bilateral GB13 improves cognitive ability via influences the levels of Aβ, p-tau (s396) and p-tau (s404) in the hippocampus of Alzheimer's disease model rats. Neuroreport. (2020) 31:1072–83. 10.1097/WNR.0000000000001518 32881772 PMC7515480

[14] Zhu M. The Medical Classic of the Yellow Emperor. (2001). Beijing: Foreign Languages Press.

[15] Wang T. Acupuncture for Brain: Treatment for Neurological and Psychologic Disorders. (2021). Cham: Springer Nature.

[16] Deng M Wang X-F. Acupuncture for amnestic mild cognitive impairment: a meta-analysis of randomised controlled trials. Acupunct Med. (2016) 34:342–8. 10.1136/acupmed-2015-010989 27491382

[17] Lu X Hongcai S Jiaying W Jing H Jun X. Assessing the quality of reports about randomized controlled trials of acupuncture treatment on mild cognitive impairment. PLoS ONE. (2011) 6:e16922. 10.1371/journal.pONE.0016922 21364920 PMC3045380

[18] Ellegaard O Wallin JA. The bibliometric analysis of scholarly production: how great is the impact? Scientometrics. (2015) 105:1809–31. 10.1007/s11192-015-1645-z 26594073 PMC4643120

[19] van Eck NJ Waltman L. Software survey: VOSviewer, a computer program for bibliometric mapping. Scientometrics. (2010) 84:523–38. 10.1007/s11192-009-0146-3 20585380 PMC2883932

[20] Chen C. CiteSpace II: Detecting and visualizing emerging trends and transient patterns in scientific literature. J Am Soc Inform Sci Technol. (2006) 57:724. 10.1002/asi.20317

[21] Chen C Hu Z Liu S Tseng H. Emerging trends in regenerative medicine: a scientometric analysis in CiteSpace. Expert Opin Biol Ther. (2012) 12:593–608. 10.1517/14712598.2012.674507 22443895

[22] Chen C. Searching for intellectual turning points: progressive knowledge domain visualization. Proc Natl Acad Sci U S A. (2004) 101 Suppl 1:5303–10. 10.1073/pnas.0307513100 14724295 PMC387312

[23] Chen CM Leydesdorff L. Patterns of Connections and Movements in Dual-Map Overlays: A New Method of Publication Portfolio Analysis. J Assoc Inf Sci Technol. (2014) 65:334–51. 10.1002/asi.22968

[24] PRICE DJdS. (1963). Little science, Big science. Columbia: Columbia University Press.

[25] Pappa S Ntella V Giannakas T Giannakoulis VG Papoutsi E Katsaounou P. Prevalence of depression, anxiety, and insomnia among healthcare workers during the COVID-19 pandemic: a systematic review and meta-analysis. Brain Behav Immun. (2020) 88:901–7. 10.1016/j.bbi.2020.05.026 32437915 PMC7206431

[26] Takeda T Hosokawa M Higuchi K. Senescence-accelerated mouse (SAM): a novel murine model of accelerated senescence. J Am Geriatr Soc. (1991) 39:911–9. 10.1111/j.1532-5415.1991.tb04460.x 1885867

[27] Takeda T Hosokawa M Takeshita S Irino M Higuchi K Matsushita T . A new murine model of accelerated senescence. Mech Ageing Dev. (1981) 17:183–94. 10.1016/0047-6374(81)90084-1 7311623

[28] Takeda T. Senescence-accelerated mouse (SAM) with special references to neurodegeneration models, SAMP8 and SAMP10 mice. Neurochem Res. (2009) 34:639–59. 10.1007/s11064-009-9922-y 19199030

[29] Jankowsky JL Fadale DJ Anderson J Xu GM Gonzales V Jenkins NA . Mutant presenilins specifically elevate the levels of the 42 residue beta-amyloid peptide in vivo: evidence for augmentation of a 42-specific gamma secretase. Hum Mol Genet. (2004) 13:159–70. 10.1093/hmg/ddh019 14645205

[30] Oakley H Cole SL Logan S Maus E Shao P Craft J . Intraneuronal beta-amyloid aggregates, neurodegeneration, and neuron loss in transgenic mice with five familial Alzheimer's disease mutations: potential factors in amyloid plaque formation. J Neurosci. (2006) 26:10129–40. 10.1523/JNEUROSCI.1202-06.2006 17021169 PMC6674618

[31] Colom LV Castaneda MT Hernandez S Perry G Jaime S Touhami A. Intrahippocampal amyloid-β (1-40) injections injure medial septal neurons in rats. Curr Alzheimer Res. (2011) 8:832–40. 10.2174/156720511798192763 22044024

[32] McLarnon JG Ryu JK. Relevance of abeta1-42 intrahippocampal injection as an animal model of inflamed Alzheimer's disease brain. Curr Alzheimer Res. (2008) 5:475–80. 10.2174/156720508785908874 18855589

[33] Farkas E Luiten PGM Bari F. Permanent, bilateral common carotid artery occlusion in the rat: a model for chronic cerebral hypoperfusion-related neurodegenerative diseases. Brain Res Rev. (2007) 54:162–80. 10.1016/j.brainresrev.2007.01.003 17296232

[34] Cheng H Yu J Jiang Z Zhang X Liu C Peng Y . Acupuncture improves cognitive deficits and regulates the brain cell proliferation of SAMP8 mice. Neurosci Lett. (2008) 432:111–6. 10.1016/j.neulet.2007.12.009 18215464

[35] Li G Zhang X Cheng H Shang X Xie H Zhang X . Acupuncture improves cognitive deficits and increases neuron density of the hippocampus in middle-aged SAMP8 mice. Acupunct Med. (2012) 30:339–45. 10.1136/acupmed-2012-010180 22975802

[36] Zhao L Zhou C Li L Liu J Shi H Kan B . Acupuncture improves cerebral microenvironment in mice with Alzheimer's disease treated with hippocampal neural stem cells. Mol Neurobiol. (2017) 54:5120–30. 10.1007/s12035-016-0054-5 27558235

[37] Zhou C-L Zhao L Shi H-Y Liu J-W Shi J-W Kan B-H . Combined acupuncture and treatment affects behavior and synaptophysin levels in the hippocampus of senescence-accelerated mouse prONE 8 after neural stem cell transplantation. Neural Regen Res. (2018) 13:541–8. 10.4103/1673-5374.228760 29623942 PMC5900520

[38] Chang S Guo X Li G Zhang X Li J Jia Y . Acupuncture promotes expression of Hsp84/86 and delays brain ageing in SAMP8 mice. Acupunct Med. (2019) 37:340–7. 10.1136/acupmed-2017-011577 31412703

[39] Zhao L Liu J-W Kan B-H Shi H-Y Yang L-P Liu X-Y. Acupuncture accelerates neural regeneration and synaptophysin production after neural stem cells transplantation in mice. World J Stem Cells. (2020) 12:1576–90. 10.4252/wjsc.v12.i12.1576 33505601 PMC7789117

[40] Li G Zeng L Cheng H Han J Zhang X Xie H. Acupuncture administration improves cognitive functions and alleviates inflammation and nuclear damage by regulating phosphatidylinositol 3 kinase (PI3K)/phosphoinositol-dependent kinase 1 (PDK1)/novel protein kinase C (nPKC)/Rac 1 signaling pathway in senescence-accelerated PrONE 8 (SAM-P8) Mice. Med Sci Monit. (2019) 25:4082–93. 10.12659/MSM.913858 31152645 PMC6559003

[41] Dong W Guo W Zheng X Wang F Chen Y Zhang W . Electroacupuncture improves cognitive deficits associated with AMPK activation in SAMP8 mice. Metab Brain Dis. (2015) 30:777–84. 10.1007/s11011-014-9641-1 25502012

[42] Dong W Quo W Wang F Li C Xie Y Zheng X . Electroacupuncture upregulates SIRT1-dependent PGC-1α expression in SAMP8 mice. Med Sci Monit. (2015) 21:3356–62. 10.12659/MSM.894864 26530101 PMC4638282

[43] Dong W-G Wang F Chen Y Zheng X-H Xie Y-C Guo W-Q . Electroacupuncture reduces Aβ production and BACE1 expression in SAMP8 mice. Front Aging Neurosci. (2015) 7:148. 10.3389/fnagi.2015.00148 26283960 PMC4518199

[44] Ding N Jiang J Xu A Tang Y Li Z. Manual acupuncture regulates behavior and cerebral blood flow in the SAMP8 mouse model of Alzheimer's disease. Front Neurosci. (2019) 13:37. 10.3389/fnins.2019.00037 30766475 PMC6365452

[45] Jiang J Liu H Wang Z Tian H Wang S Yang J . Electroacupuncture could balance the gut microbiota and improve the learning and memory abilities of Alzheimer's disease animal model. PLoS ONE. (2021) 16:e0259530. 10.1371/journal.pONE.0259530 34748592 PMC8575259

[46] Liang P-Z Li L Zhang Y-N Shen Y Zhang L-L Zhou J . Electroacupuncture improves clearance of amyloid- through the glymphatic system in the SAMP8 mouse model of Alzheimer's disease. Neural Plast. (2021) 2021:9960304. 10.1155/2021/9960304 34484327 PMC8416373

[47] Jiang J Liu G Shi S Li Y Li Z. Effects of manual acupuncture combined with dONEpezil in a mouse model of Alzheimer's disease. Acupunct Med. (2019) 37:64–71. 10.1136/acupmed-2016-011310 30843424

[48] Dong W Yang W Li F Guo W Qian C Wang F . Electroacupuncture improves synaptic function in SAMP8 mice probably via inhibition of the AMPK/eEF2K/eEF2 signaling pathway. Evid Based Complement Alternat Med. (2019) 2019:8260815. 10.1155/2019/8260815 31641367 PMC6766673

[49] Hou Z Qiu R Wei Q Liu Y Wang M Mei T . Electroacupuncture improves cognitive function in senescence-accelerated P8 (SAMP8) mice via the NLRP3/caspase-1 pathway. Neural Plast. (2020) 2020:8853720. 10.1155/2020/8853720 33204250 PMC7657681

[50] Wang X Li Z Li C Wang Y Yu S Ren L. Electroacupuncture with Bushen Jiannao improves cognitive deficits in senescence-accelerated mouse prONE 8 mice by inhibiting neuroinflammation. J Tradit Chin Med. (2020) 40:812–9. 10.19852/j.cnki.jtcm.2020.05.011 33000582

[51] Li X Guo F Zhang Q Huo T Liu L Wei H . Electroacupuncture decreases cognitive impairment and promotes neurogenesis in the APP/PS1 transgenic mice. BMC Complement Altern Med. (2014) 14:37. 10.1186/1472-6882-14-37 24447795 PMC3907495

[52] Lin R Li L Zhang Y Huang S Chen S Shi J . Electroacupuncture ameliorate learning and memory by improving N-acetylaspartate and glutamate metabolism in APP/PS1 mice. Biol Res. (2018) 51:21. 10.1186/s40659-018-0166-7 29980225 PMC6034239

[53] Liu W Zhuo P Li L Jin H Lin B Zhang Y . Activation of brain glucose metabolism ameliorating cognitive impairment in APP/PS1 transgenic mice by electroacupuncture. Free Radic Biol Med. (2017) 112:174–90. 10.1016/j.freeradbiomed.2017.07.024 28756309

[54] Wang F Zhong H Li X Peng Y Kinden R Liang W . Electroacupuncture attenuates reference memory impairment associated with astrocytic NDRG2 suppression in APP/PS1 transgenic mice. Mol Neurobiol. (2014) 50:305–13. 10.1007/s12035-013-8609-1 24390566

[55] Lin R Chen J Li X Mao J Wu Y Zhuo P . Electroacupuncture at the Baihui acupoint alleviates cognitive impairment and exerts neuroprotective effects by modulating the expression and processing of brain-derived neurotrophic factor in APP/PS1 transgenic mice. Mol Med Rep. (2016) 13:1611–7. 10.3892/mmr.2015.4751 26739187 PMC4732857

[56] Cao J Tang Y Li Y Gao K Shi X Li Z. Behavioral changes and hippocampus glucose metabolism in APP/PS1 transgenic mice via electro-acupuncture at governor vessel acupoints. Front Aging Neurosci. (2017) 9:5. 10.3389/fnagi.2017.00005 28174534 PMC5259686

[57] Xu A Tang Y Zeng Q Wang X Tian H Zhou Y . Electroacupuncture enhances cognition by promoting brain glucose metabolism and inhibiting inflammation in the APP/PS1 mouse model of Alzheimer's disease: a pilot study. J Alzheimers Dis. (2020) 77:387–400. 10.3233/JAD-200242 32741819

[58] Tang Y Shao S Guo Y Zhou Y Cao J Xu A . Electroacupuncture mitigates hippocampal cognitive impairments by reducing BACE1 deposition and activating PKA in APP/PS1 double transgenic mice. Neural Plast. (2019) 2019:2823679. 10.1155/2019/2823679 31223308 PMC6541940

[59] Tang Y Xu A Shao S Zhou Y Xiong B Li Z. Electroacupuncture ameliorates cognitive impairment by inhibiting the JNK signaling pathway in a mouse model of Alzheimer's disease. Front Aging Neurosci. (2020) 12:23. 10.3389/fnagi.2020.00023 32116652 PMC7016202

[60] Xu A Zeng Q Tang Y Wang X Yuan X Zhou Y . Electroacupuncture protects cognition by regulating tau phosphorylation and glucose metabolism via the AKT/GSK3β signaling pathway in Alzheimer's disease model mice. Front Neurosci. (2020) 14:585476. 10.3389/fnins.2020.585476 33328854 PMC7714768

[61] Yang Q Zhu S Xu J Tang C Wu K Wu Y . Effect of the electro-acupuncture on senile plaques and its formation in APP/PS1 double transgenic mice. Genes Dis. (2019) 6:282–9. 10.1016/j.gendis.2018.06.002 32042867 PMC6997572

[62] Li J Zhang B Jia W Yang M Zhang Y Zhang J . Activation of adenosine monophosphate-activated protein kinase drives the aerobic glycolysis in hippocampus for delaying cognitive decline following electroacupuncture treatment in APP/PS1 mice. Front Cell Neurosci. (2021) 15:774569. 10.3389/fncel.2021.774569 34867206 PMC8636716

[63] Cai M Yang EJ. Effect of Combined Electroacupuncture and selegiline treatment in Alzheimer's disease: an animal model. Front Pharmacol. (2020) 11:606480. 10.3389/fphar.2020.606480 33362561 PMC7758426

[64] Cai M Lee J-H Yang EJ. Electroacupuncture attenuates cognition impairment via anti-neuroinflammation in an Alzheimer's disease animal model. J Neuroinflammation. (2019) 16:264. 10.1186/s12974-019-1665-3 31836020 PMC6909515

[65] Zheng X Lin W Jiang Y Lu K Wei W Huo Q . Electroacupuncture ameliorates beta-amyloid pathology and cognitive impairment in Alzheimer disease via a novel mechanism involving activation of TFEB (transcription factor EB). Autophagy. (2021) 17:3833–47. 10.1080/15548627.2021.1886720 33622188 PMC8632298

[66] Li L Li J Dai Y Yang M Liang S Wang Z . Electro-acupuncture improve the early pattern separation in Alzheimer's disease mice basal forebrain-hippocampus cholinergic neural circuit. Front Aging Neurosci. (2021) 13:770948. 10.3389/fnagi.2021.770948 35185516 PMC8847781

[67] Zhang M Xv G-H Wang W-X Meng D-J Ji Y. Electroacupuncture improves cognitive deficits and activates PPAR-γ in a rat model of Alzheimer's disease. Acupunct Med. (2017) 35:44–51. 10.1136/acupmed-2015-010972 27401747

[68] Guo H-D Tian J-X Zhu J Li L Sun K Shao S-J . Electroacupuncture suppressed neuronal apoptosis and improved cognitive impairment in the AD model rats possibly via downregulation of notch signaling pathway. Evid Based Complement Alternat Med. (2015) 2015:393569. 10.1155/2015/393569 25810743 PMC4355557

[69] Yu C-C Wang Y Shen F Kong L-H Wang Y-W Zhou H . High-frequency (50 Hz) electroacupuncture ameliorates cognitive impairment in rats with amyloid beta 1-42-induced Alzheimer's disease. Neural Regen Res. (2018) 13:1833–41. 10.4103/1673-5374.238620 30136700 PMC6128060

[70] Wang Y Zheng A Yang H Wang Q Ren B Guo T . “Olfactory three-needle” acupuncture enhances synaptic function in Aβ-induced Alzheimer's disease via activating PI3K/AKT/GSK-3β signaling pathway. J Integr Neurosci. (2021) 20:55–65. 10.31083/j.jin.2021.01.224 33834691

[71] Wang X-R Shi G-X Yang J-W Yan C-Q Lin L-T Du S-Q . Acupuncture ameliorates cognitive impairment and hippocampus neuronal loss in experimental vascular dementia through Nrf2-mediated antioxidant response. Free Radic Biol Med. (2015) 89:1077–84. 10.1016/j.freeradbiomed.2015.10.426 26546103

[72] Zhu W Wang X-R Du S-Q Yan C-Q Yang N-N Lin L-L . Anti-oxidative and Anti-apoptotic Effects of Acupuncture: Role of Thioredoxin-1 in the Hippocampus of Vascular Dementia Rats. Neuroscience. (2018) 379:281–91. 10.1016/j.neuroscience.2018.03.029 29592844

[73] Du S-Q Wang X-R Zhu W Ye Y Yang J-W Ma S-M . Acupuncture inhibits TXNIP-associated oxidative stress and inflammation to attenuate cognitive impairment in vascular dementia rats. CNS Neurosci Ther. (2018) 24:39–46. 10.1111/cns.12773 29110407 PMC6489958

[74] Qiu J Shen B Zhao M Wang Z Xie B Xu Y . nationwide survey of psychological distress among Chinese people in the COVID-19 epidemic: implications and policy recommendations. Gen Psychiatr. (2020) 33:e100213. 10.1136/gpsych-2020-100213 32215365 PMC7061893

[75] Ye Y Li H Yang J-W Wang X-R Shi G-X Yan C-Q . Acupuncture attenuated vascular dementia-induced hippocampal long-term potentiation impairments via activation of D1/D5 receptors. Stroke. (2017) 48:1044–51. 10.1161/STROKEAHA.116.014696 28289242

[76] Li H Liu Y Lin L-T Wang X-R Du S-Q Yan C-Q . Acupuncture reversed hippocampal mitochondrial dysfunction in vascular dementia rats. Neurochem Int. (2016) 92:35–42. 10.1016/j.neuint.2015.12.001 26682902

[77] Kang L Li Y Hu S Chen M Yang C Yang BX . The mental health of medical workers in Wuhan, China dealing with the 2019 novel coronavirus. Lancet Psychiatry. (2020) 7:e14. 10.1016/S2215-0366(20)30047-X 32035030 PMC7129673

[78] Yang J-W Wang X-R Zhang M Xiao L-Y Zhu W Ji C-S . Acupuncture as a multifunctional neuroprotective therapy ameliorates cognitive impairment in a rat model of vascular dementia: a quantitative iTRAQ proteomics study. CNS Neurosci Ther. (2018) 24:1264–74. 10.1111/cns.13063 30278105 PMC6490085

[79] Zhu Y Zeng Y Wang X Ye X. Effect of electroacupuncture on the expression of mTOR and eIF4E in hippocampus of rats with vascular dementia. Neurol Sci. (2013) 34:1093–7. 10.1007/s10072-012-1209-4 23053837

[80] Zhu Y Wu Q Lin L. Effects of electro-acupuncture on Noxa and caspase-3 expression in hippocampal CA1 region of a vascular dementia rat model. Neural Regener Res. (2008) 3:826–31.

[81] Zhu Y Zeng Y. Electroacupuncture protected pyramidal cells in hippocampal CA1 region of vascular dementia rats by inhibiting the expression of p53 and Noxa. CNS Neurosci Ther. (2011) 17:599–604. 10.1111/j.1755-5949.2010.00192.x 20950325 PMC6493847

[82] Wei D Jia X Yin X Jiang W. Effects of electroacupuncture versus nimodipine on long-term potentiation and synaptophysin expression in a rat model of vascular dementia. Neural Regener Res. (2011) 6:2357–61. 10.3969/j.issn.1673-5374.2011.30.007

[83] Bu Y Li W-S Lin J Wei Y-W Sun Q-Y Zhu S-J . Electroacupuncture attenuates immune-inflammatory response in hippocampus of rats with vascular dementia by inhibiting TLR4/MyD88 signaling pathway. Chin J Integr Med. (2022) 28:153–61. 10.1007/s11655-021-3350-5 34913150 PMC8672855

[84] Zhu Y Wang X Ye X Gao C Wang W. Effects of electroacupuncture on the expression of p70 ribosomal protein S6 kinase and ribosomal protein S6 in the hippocampus of rats with vascular dementia. Neural Regener Res. (2012) 7:207–11. 10.3969/j.issn.1673-5374.2012.03.009 25767501 PMC4353116

[85] Xiao Q Yan P Ma X Liu H Perez R Zhu A . Neuronal-targeted TFEB accelerates lysosomal degradation of APP, reducing Aβ generation and amyloid plaque pathogenesis. J Neurosci. (2015) 35:12137–51. 10.1523/JNEUROSCI.0705-15.2015 26338325 PMC4556784

[86] Feng Y Bai L Ren Y Chen S Wang H Zhang W . FMRI connectivity analysis of acupuncture effects on the whole brain network in mild cognitive impairment patients. Magn Reson Imaging. (2012) 30:672–82. 10.1016/j.mri.2012.01.003 22459434

[87] Zheng W Su Z Liu X Zhang H Han Y Song H . Modulation of functional activity and connectivity by acupuncture in patients with Alzheimer disease as measured by resting-state fMRI. PLoS ONE. (2018) 13:e0196933. 10.1371/journal.pONE.0196933 29763448 PMC5953467

[88] Chen S Bai L Xu M Wang F Yin L Peng X . Multivariate granger causality analysis of acupuncture effects in mild cognitive impairment patients: an FMRI study. Evid Based Complement Alternat Med. (2013) 2013:127271. 10.1155/2013/127271 24023568 PMC3760118

[89] Yan W-T Yang Y-D Hu X-M Ning W-Y Liao L-S Lu S . Do pyroptosis, apoptosis, and necroptosis (PANoptosis) exist in cerebral ischemia? Evidence from cell and rodent studies. Neural Regen Res. (2022) 17:1761–8. 10.4103/1673-5374.331539 35017436 PMC8820688

[90] Chen Y Li Y Guo L Hong J Zhao W Hu X . Bibliometric analysis of the inflammasome and pyroptosis in brain. Front Pharmacol. (2020) 11:626502. 10.3389/fphar.2020.626502 33551822 PMC7854385

[91] Yan W-T Lu S Yang Y-D Ning W-Y Cai Y Hu X-M . Research trends, hot spots and prospects for necroptosis in the field of neuroscience. Neural Regen Res. (2021) 16:1628–37. 10.4103/1673-5374.303032 33433494 PMC8323674

